# Proteomic Profiling Reveals Cytotoxic Mechanisms of Action and Adaptive Mechanisms of Resistance in *Porphyromonas gingivalis*: Treatment with *Juglans regia* and *Melaleuca alternifolia*

**DOI:** 10.1021/acsomega.3c00168

**Published:** 2023-03-28

**Authors:** Afrah E. Mohammed, Kawther Aabed, Hicham Benabdelkamel, Ashwag Shami, Modhi O. Alotaibi, Mona Alanazi, Assim A. Alfadda, Ishrat Rahman

**Affiliations:** † Department of Biology, College of Science, Princess Nourah bint Abdulrahman University, P.O. Box 84428, Riyadh 11671, Saudi Arabia; ‡ Proteomics Resource Unit, Obesity Research Center, College of Medicine, King Saud University, P.O. Box 2925 (98), Riyadh 11461, Saudi Arabia; § Department of Basic Dental Sciences, College of Dentistry, Princess Nourah bint Abdulrahman University, P.O. Box 84428, Riyadh 11671, Saudi Arabia; ¶ Department of Medicine, College of Medicine and King Saud Medical City, King Saud University, P.O. Box 2925 (98), Riyadh 11461, Saudi Arabia

## Abstract

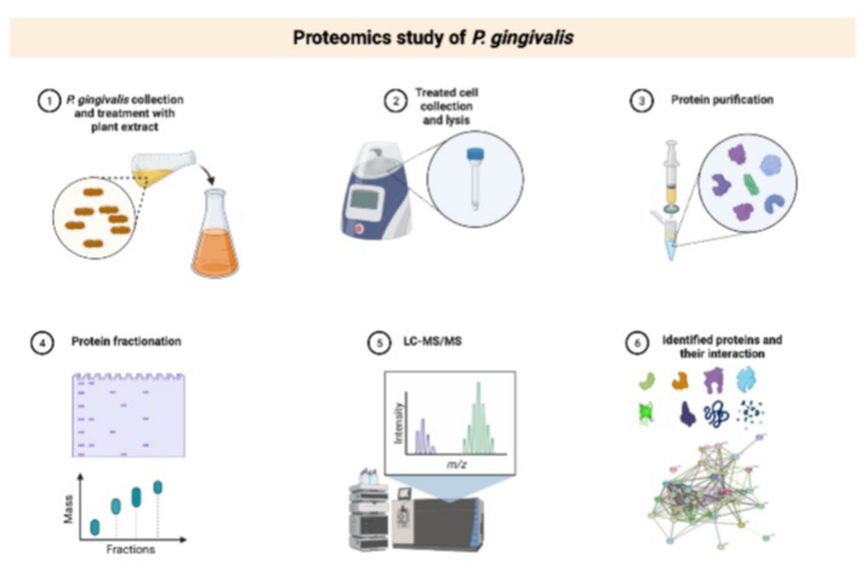

The increasing trend in the rise of antibiotic-resistant bacteria pushes research to discover new efficacious antibacterial agents from natural and synthetic sources. *Porphyromonas gingivalis* is a well-known bacterium commonly known for causing periodontal disease, and it is associated with the pathogenesis of life-changing systemic conditions such as Alzheimer’s. Proteomic research can be utilized to test new antibacterial drugs and understand the adaptive resistive mechanisms of bacteria; hence, it is important in the drug discovery process. The current study focuses on identifying the antibacterial effects of *Juglans regia* (JR) and *Melaleuca alternifolia* (MA) on *P. gingivalis* and uses proteomics to identify modes of action while exploring its adaptive mechanisms. JR and MA extracts were tested for antibacterial efficacy using the agar well diffusion assay. A proteomic study was conducted identifying upregulated and downregulated proteins compared to control by 2D-DIGE analysis, and proteins were identified using MADLI-TOF/MS. The bacterial inhibition for JR was 20.14 ± 0.2, and that for MA was 19.72 ± 0.5 mm. Out of 88 differentially expressed proteins, there were 17 common differentially expressed proteins: 10 were upregulated and 7 were downregulated in both treatments. Among the upregulated proteins were Arginine-tRNA ligase, ATP-dependent Clp protease proteolytic, and flavodoxins. In contrast, down-regulated proteins were ATP synthase subunit alpha and quinone, among others, which are known antibacterial targets. STRING analysis indicated a strong network of interactions between differentially expressed proteins, mainly involved in protein translation, post-translational modification, energy production, metabolic pathways, and protein repair and degradation. Both extracts were equi-efficacious at inhibiting *P. gingivalis* and displayed some overlapping proteomic profiles. However, the MR extract had a greater fold change in its profile than the JA extract. Downregulated proteins indicated similarity in the mode of action, and upregulated proteins appear to be related to adaptive mechanisms important in promoting repair, growth, survival, virulence, and resistance. Hence, both extracts may be useful in preventing *P. gingivalis*-associated conditions. Furthermore, our results may be helpful to researchers in identifying new antibiotics which may offset these mechanisms of resistance.

## 1 Introduction

The rampant use of antibiotics globally has led to an increase in bacterial resistance and therefore poses a significant risk to human health. As such, it is a priority for researchers to identify novel compounds which are safe and efficacious against pathogenic bacteria. Some of the most commonly encountered infections arise from the oral cavity. These infections are mostly mild, easily preventable, and treatable; however, in immunocompromised patients, such infections can lead to serious health outcomes.^1,2^ In addition, there is also increasing evidence that the pathogenesis of many severe conditions, such as Alzheimer’s and cardiovascular diseases, may be related to infections arising from the oral cavity.^3^*Porphyromonas gingivalis* is an oral pathogenic anaerobic Gram-negative bacterium that infects and damages tooth enamel and promotes severe inflammation of the periodontal tissues by affecting the host defense mechanisms, ultimately causing tooth loss.^4^ Interestingly, even biotechs and pharmaceuticals are keen to target secreted proteins from *P. gingivalis*, such as gingipains, to prevent the pathogenesis of Alzheimer’s.^5^ In addition, well-known antibiotics such as macrolides, clindamycin, and tetracyclines are ineffective in *P. gingivalis*-associated infections attributed to the switching on of specific resistance genes, *erm*(*B*), *erm*(*F*), and *tet*(*Q*).^6^ It is important to note that most strains of *P. gingivalis* are susceptible to amoxicillin and metronidazole and are not resistant against amoxicillin + clavulanic acid and the cephalosporin drug, cefoxitin, which are the primary antibiotics used by dental professionals for periodontal infections.^6^ However, since bacteria are ever evolving to survive antimicrobials and the rate of bacterial resistance against well-known antibiotics increases yearly, it is necessary to preemptively develop novel, safer, and more efficacious antibiotics.

Natural products interest researchers because of their therapeutic effects against many diseases, attributed to the diversity in secondary metabolites. In addition, various natural phytochemicals are recognized as antibacterial^7^ and anticancer agents.^8^ Plants are a major source of novel compounds with antibiotic and anti-inflammatory effects. Currently, using plants as a source of compounds for the treatment of infections is gaining interest from researchers worldwide due to the potentially safer toxicity profile in comparison to traditional chemically synthesized compounds. Although herbs and their phytochemicals are generally accepted as a safer, cost-effective, and relatively efficacious way of treating various infections, far less is known about the mode of action and their targets within the bacterial metabolic pathways and enzymes. In addition, some studies have explored the effect of antimicrobial compounds on bacterial gene expression profiles to identify mechanisms of bacterial resistance.^9^

*Juglans regia* (family Juglandaceae), also known as English Walnut, is known for its therapeutic effects as an anti-inflammatory, depurative, anticancer, laxative, blood purifying, and antimicrobial agent.^10^ The stem of *J. regia* contains active phytochemicals such as gallic acid, folic acid, ascorbic acid, 5 juglone, β-sitosterol, quercetin-3-α-L-arabinoside, regiolone, and polyphenol.^11^*Melaleuca alternifolia*, known as tea tree, is naturally found in Australia, and tea tree oil is available in markets worldwide. It is extracted by the steam concentration of the *M. alternifolia* leaves, and it contains an abundance of phytochemicals appreciated for its analgesic, antifungal, antimicrobial, and anti-inflammatory properties.^12^ Tea tree oil is utilized in the cosmetic and healthcare industry as a natural and safer antiseptic.^13^ Terpinen-4-ol and α-terpineol are key antimicrobial metabolites in tea tree oil.^14^ The application of proteomics is an ideal area of focus to understand the mechanisms of action of antimicrobials and the responsive mechanisms of bacterial adaptation and resistance. In addition, it helps achieve precise drug targets to combat resistant and deadly bacterial infection and can be utilized for measuring the efficacy of antimicrobial drugs, which include phytochemicals.^15^ Proteomics study using 2D gel electrophoresis can also contribute valuable information with high-resolution profiling of low-abundance proteins.

Several studies have identified the cytotoxic effect of *J. regia* and *M. alternifolia* on *P. gingivalis*.^16,17^ However, the current study is the first to report on the proteomic profile of *P. gingivalis* following exposure to the extracts. We aim to identify the mode of action of these cytotoxic herbal extracts and decipher the responsive mechanisms of the bacteria, identifying targets that may help researchers to develop novel and more efficacious antimicrobials impacting oral health and systemic diseases associated with *P. gingivalis* infection.

## 2 Methodology

### 2.1 Plant Materials

*J. regia* (JR) and the oil of *M. alternifolia* (MA) were obtained from a local market in Riyadh, Saudi Arabia. JR leaves were rinsed with distilled water and then air-dried and milled using a milling device (IKA Werke Laboratory Equipment, Staufen, Germany). The powder was stored at room temperature in a sealed plastic box for further investigation.

### 2.2 Preparation of the Extracts

Aqueous extracts were prepared from MA by adding 2 mL of tea tree oil to 100 mL of distilled water and 2 g of JR milled leaves to 100 mL of distilled water. Solutions were heated for 15 min at 80 °C and filtered through Whatman candidate No. 1 (pore size 125 mm, Whatman, Maidstone, UK). The aqueous filtrate was stored at 4 °C.

### 2.3 Antimicrobial Effect

The antibacterial effect of JR, MA, amoxicillin, and chlorohexidine were tested against *Porphyromonas gingivalis* using an agar well diffusion assay.^18^ Distilled water was used as a negative control.

### 2.4 Protein Extraction

Protein extraction was carried out from *P. gingivalis* cells (control and treated) as previously reported.^19^ Briefly, the cells were centrifuged for 5 min at 5,000*g* at 4 °C. Then, the supernatants were discarded, and the resulting pellets were washed with phosphate-buffered saline (PBS). Subsequently, the protein pellets were suspended in lysis buffer (0.5 mL; pH 8.8; 30 mM Tris buffer containing 7 M urea, 2 M thiourea, 2% Chaps, and the protease inhibitor cocktail; GE Healthcare, Chicago, IL, USA) on ice for 20 min. After that, samples were vortexed and sonicated (3–4 times) for 1 min and recentrifuged at 10,000*g* at 4 °C for 3 min to remove cell debris. Finally, protein concentrations were determined using the 2D-Quant Kit according to the manufacturer’s instructions (GE Healthcare).

### 2.5 Labeling of Protein and Two-Dimensional (2D) Gel Electrophoresis

50 μg of total protein per sample was covalently labeled with a fluorophore, either Cy3 or Cy5, by adding 400 pmol of CyDyes (DIGE Fluor dyes, GE Healthcare, UK) in 1 μL of dimethylmethanamide (DMF), and then incubating for 30 min on ice. To terminate the labeling reaction, 1 μL of 10 mM lysine was added to each sample. In addition, an equal amount of all samples in the experiment was pooled as an internal standard and labeled with Cy2. Two-dimensional analysis gel electrophoresis was performed as described by Alfadda et al.^20^ Briefly, 1 mg of total protein from the pool was added to the rehydration buffer (7 M urea, 2 M thiourea, 4% CHAPS, 0.006 g DTT, 2 μL of bromophenol blue, 5 μL of IPG buffer (pI 3–11), 1× protease inhibitor mix) applied to 5 Immobiline Dry Strips (24 cm, pH 3–11; GE Healthcare, Sweden). Isoelectric focusing was performed at 50 μA per strip using an Ettan IPGphor IEF unit (GE Healthcare, Sweden, 30 V, 12h, 20 °C). Soon after, the strips were equilibrated and separated on 12.5% (SDS-PAGE) gels using an Ettan Dalt Six device (GE Healthcare, Sweden). The gels were scanned with the appropriate wavelengths (Cy2, 488/520 nm; Cy3, 32/580 nm; and Cy5, 633/670 nm) and filters for the CyDyes dyes using a Typhoon 9400 scanner (GE Healthcare, Chicago, IL, USA).

### 2.6 Matrix-Assisted Laser Desorption/Ionization Time-of-Flight (MALDI-TOF) Mass Spectrometry (MS) for Protein Identification

A preparative gel from total protein (1 mg) was prepared and obtained with samples from a pool of equal protein amounts from nine samples (triplicates from control, JR, and MA), as reported previously.^19^ Afterward, the Coomassie blue stained gel spots were excised manually into a 96-well plate and washed. They were then digested by adding ice-cold trypsin solution consisting of 20 ng of sequencing grade modified porcine trypsin (Promega, USA) in 25 mM NH_4_HCO_3_ (pH 8.0) for 20 min at 4 °C. Digestion continued overnight at 37 °C. Subsequently, 1 uL of 1% trifluoracetic acid was added to the gel pieces to stop the reaction. Peptides were extracted by placing the samples in a vortex incubator for 1 h at 400 rpm at 25 °C. Finally, 0.8 μL of tryptic peptides’ mixture derived from each Coomassie protein spot was spotted onto a MALDI target (384 MTP Anchorchip; 800 μm Anchorchip; Bruker Daltonics, Bremen, Germany). MALDI-MS(/MS) spectra were obtained, and peptide mass fingerprints (PMFs) were identified using an UltraflexTerm time-of-flight (TOF) mass spectrometer equipped with a LIFT-MS/MS device (Bruker Daltonics) at reflector and detector voltages of 21 and 17 kV, respectively.^19−22^ PMFs were calibrated against a standard (peptide calibration standard II, Bruker Daltonics) and were evaluated using the Flex Analysis software (version 2.4, Bruker Daltonics). MS data was constructed using the BioTools v3.2 (Bruker Daltonics) software. The peptide masses were identified using the Mascot search algorithm (v2.0.04, updated on 09/05/2020; Matrix Science Ltd., UK). The identified proteins were differentiated based on a Mascot score of higher than 56 and *p* < 0.05.

### 2.7 Statistical Analysis

The antibacterial results from the agar well diffusion assay were analyzed using GraphPad prism, and the data are presented as the mean and standard deviation for three replicate experiments. Progenesis SameSpots software (Nonlinear Dynamics, UK) was used for 2D-DIGE gel image analysis using an automated spot detection method. The analysis involved comparing control, MA-treated, and JR-treated samples. ANOVA tests were performed for statistical analysis of the 2D-DIGE gel images. Furthermore, automated analysis was used for spot detection in all gels. Each spot was confirmed and edited manually where necessary. Values were normalized to detect the differentially expressed spots. A cutoff ratio ≥1.5-fold was considered significant.

### 2.8 Bioinformatics Analysis and Protein Functional Classification

The STRING database determines the pathways and functions most closely related to identified proteins by overlaying and correlating input results with trial expression data on networks built from reported interactions. Obtained quantitative data was uploaded onto STRING v11.0 (https://string-db.org/) to analyze protein networks.

## 3 Results

### 3.1 Antibacterial Assessment

The aqueous extracts from JR and MA showed antibacterial activity against *P. Gingivalis*, with inhibition of 20.14 ± 0.2 and 19.72 ± 0.5 mm, respectively, identifying no significant variation in activity between the extracts (*p* ≤ 0.33). JR and MA extract activities were significantly higher than the activity of amoxicillin (16.23 ± 0.3 mm) at *p* ≤ 0.005 and *p* ≤ 0.05, respectively, and lower than that of chlorohexidine (24.58 ± 0.9 mm).

### 3.2 2D-DIGE Assessment and Proteomic Study

**Figure 1:**
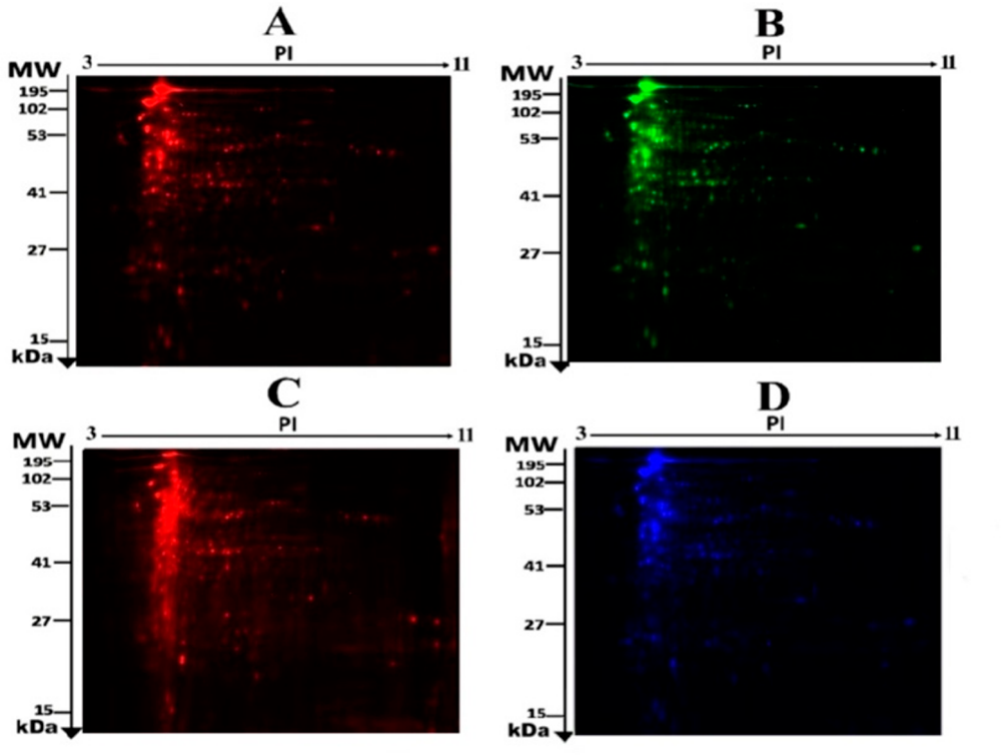
Fluorescent proteins of a two-dimensional difference in gel electrophoresis (2D-DIGE) having control, Cy5 (A); JR-treated, Cy3 (B); MA-treated, Cy 5 (C); and pooled labeled with Cy2 (D).

**Figure 2:**
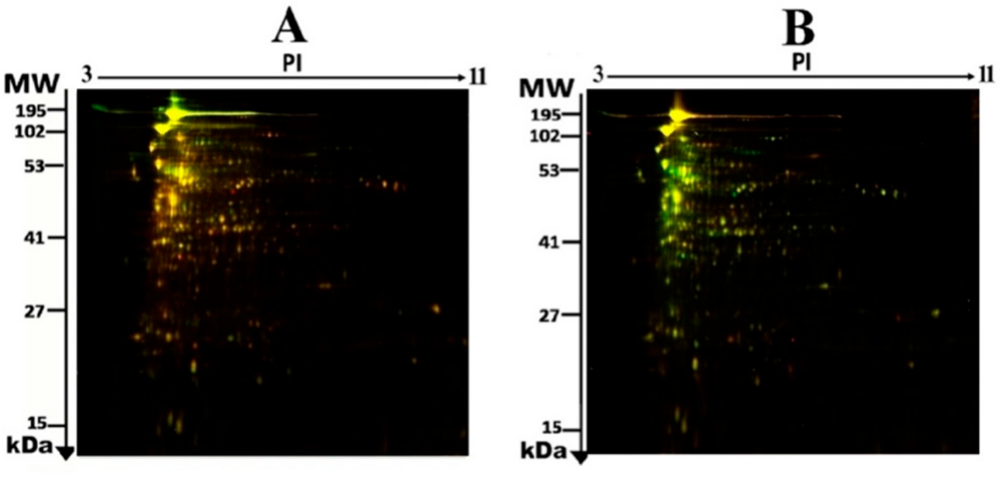
Two-dimensional difference in gel electrophoresis (2D-DIGE) representative fluorescent protein that involves merged samples from control and JR-treated (A) and control and MA-treated (B).

**Figure 3:**
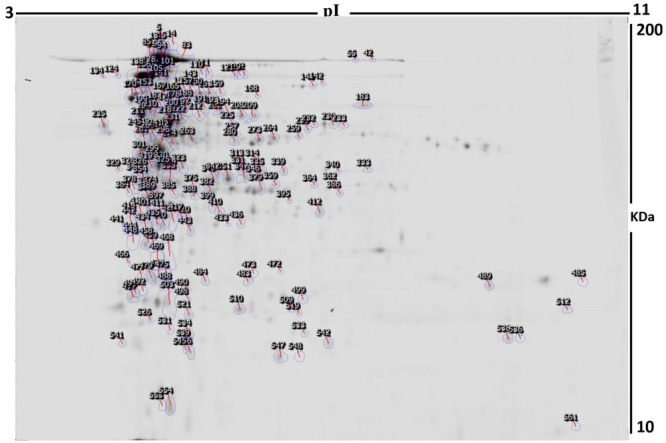
2D-DIGE numbered spots show differentially abundant proteins (defined as fold-change >1.5, *p* < 0.05) between the control, JR-treated, and MA-treated samples (MS). MW, protein molecular weight; pI, isoelectric point.

Results from the 2D-DIGE displaying fluorescent protein profiles are shown in Figure 1. Untreated control is labeled with Cy5 (Figure 1A), JR-treated labeled with Cy3 (Figure 1B), MA-treated labeled with Cy5 (Figure 1C), and pooled internal control labeled with Cy2 (Figure 1D). The Merged 2D-DIGE gels of control with JR-treated and control with MA-treated labeled with Cy3/Cy5 are presented in Figure 2. A significant change in protein abundance levels (fold-change ≥ 1.5) was observed in JR-treated (Figure 2A) and MA-treated (Figure 2B) samples among the identified spots on the gels compared with the control (*p* ≤ 0.05). The quantitative differential analysis of the protein levels and normalization within all gels were evaluated by comparing the internal standard (pooled sample) with Cy2-labeling. The Progenesis SameSpot statistical software identified a total of 190 spots that indicated a significant increment or decrement in protein expression from the preparative gel (Figure 3) for further protein identification by MS.

Using MALDI-TOF mass spectrometry, 88 proteins were identified (listed in Table 1) out of the 190 differentially expressed protein spots observed in the pooled sample or preparative gel (Figure 3). In addition, peptide mass fingerprints (PMFs) identified 75 out of 88 spots as protein sequences corresponding to entries in the SWISS-PROT database with high Mascot confidence scores (>56) (*p* < 0.05) (Table 1 and Table S2).

**Table 1:**
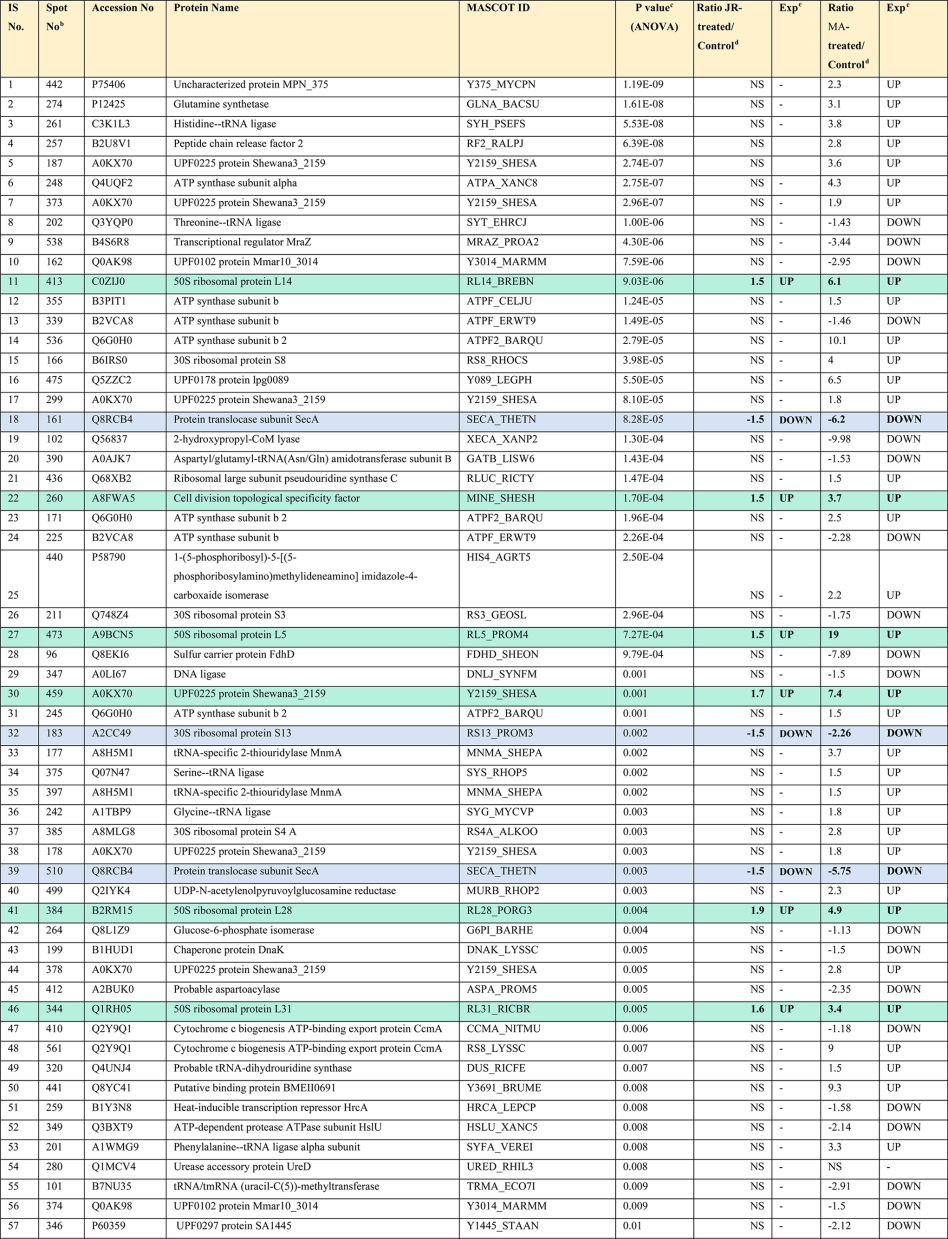

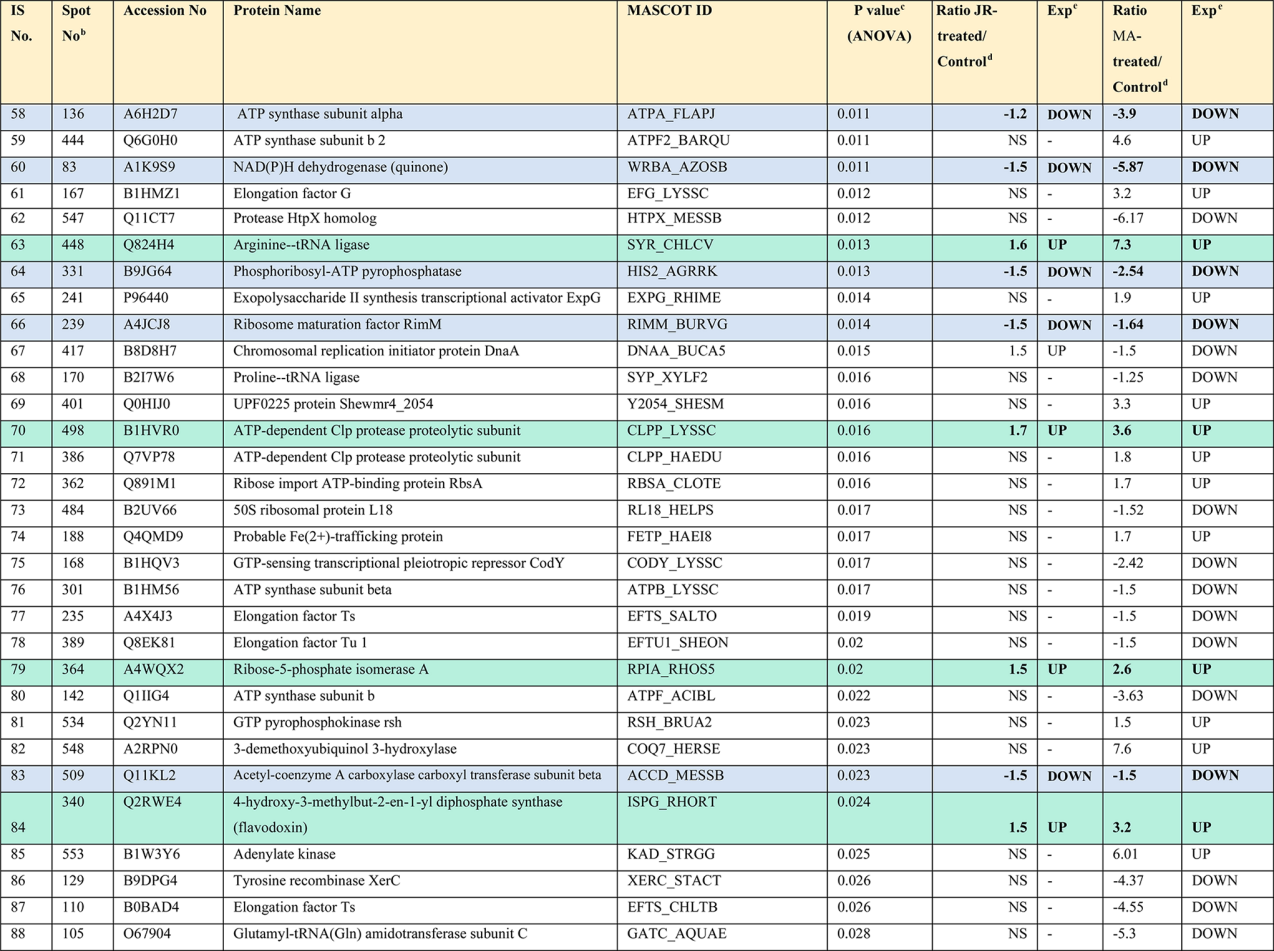
Differentially Expressed Proteins and Their Abundance Changes among Control, JR-Treated, and MA-Treated Samples^a^

a Accession number, protein name, Mascot score, and one-way ANOVA (p-value <0.05). Data derived from the original 2D-DIGE gels were analyzed to determine the mean ratio between the treatments and their corresponding levels of fold changes [Analysis type: MALDI-TOF; database: SwissProt; taxonomy: Bacteria]. Commonly upregulated proteins are highlighted in green, and commonly downregulated proteins are highlighted in blue.

b Protein accession number for SWISSPROT Database.

c p-Value (ANOVA).

d Ratio between the groups.

e Protein expression between the groups.

From the total of 88 proteins identified in the pooled sample, in JR-treated bacteria, only 11 proteins were upregulated and 8 were downregulated, whereas 69 proteins had no change in expression (not significantly different from the control) (Table 1, Figure 3). Among them, the most significantly upregulated proteins included 50s ribosomal protein L14 (up 1.5-fold, *p* = 9.03 × 10^–6^) and 4-hydroxy-3-methylbut-2-en-1-yl diphosphate synthase (flavodoxin) (up 1.5-fold, *p* = 0.024), and the most significantly downregulated proteins included protein translocase subunit Sec A (down 1.5 fold, *p* = 8.28 × 10^–5^) and 30S ribosomal protein S13 (down 1.5 fold, *p* = 0.002). However, in MA-treated bacteria, 48 protein spots were upregulated, 38 were downregulated, and only 2 proteins had no significant change in expression compared to the control. Among them, the most significantly upregulated proteins included 50s ribosomal protein L5 (up 19-fold, *p* = 7.27 × 10^–4^) and ATP synthase subunit b2 (up 10.1-fold, *p* = 2.79 × 10^–5^). On the other hand, the significantly downregulated proteins included 2-hydroxypropyl-CoM lyase (down 9.98-fold, *p* = 1.30 × 10^–4^) and sulfur carrier protein FdhD (down 7.89-fold, *p* = 9.79 × 10^–4^). The complete list of upregulated and downregulated proteins in each treatment is shown in Table 1 and Table S2.

In some incidents, the same protein variants were detected at various locations on the gel (Table S2 and Figure 3). Those identified proteins were UPF0225 protein Shewana3_2159, UPF0102 protein Mmar10_3014, ATP synthase subunit b, ATP synthase subunit b2, protein translocase subunit SecA, tRNA-specific 2-thiouridylase MnmA, Cytochrome c biogenesis ATP-binding export protein CcmA, elongation factor Ts, and ATP-dependent Clp protease proteolytic subunit.

### 3.3 Principal Component, Cluster Analysis, and Heatmap

**Figure 4:**
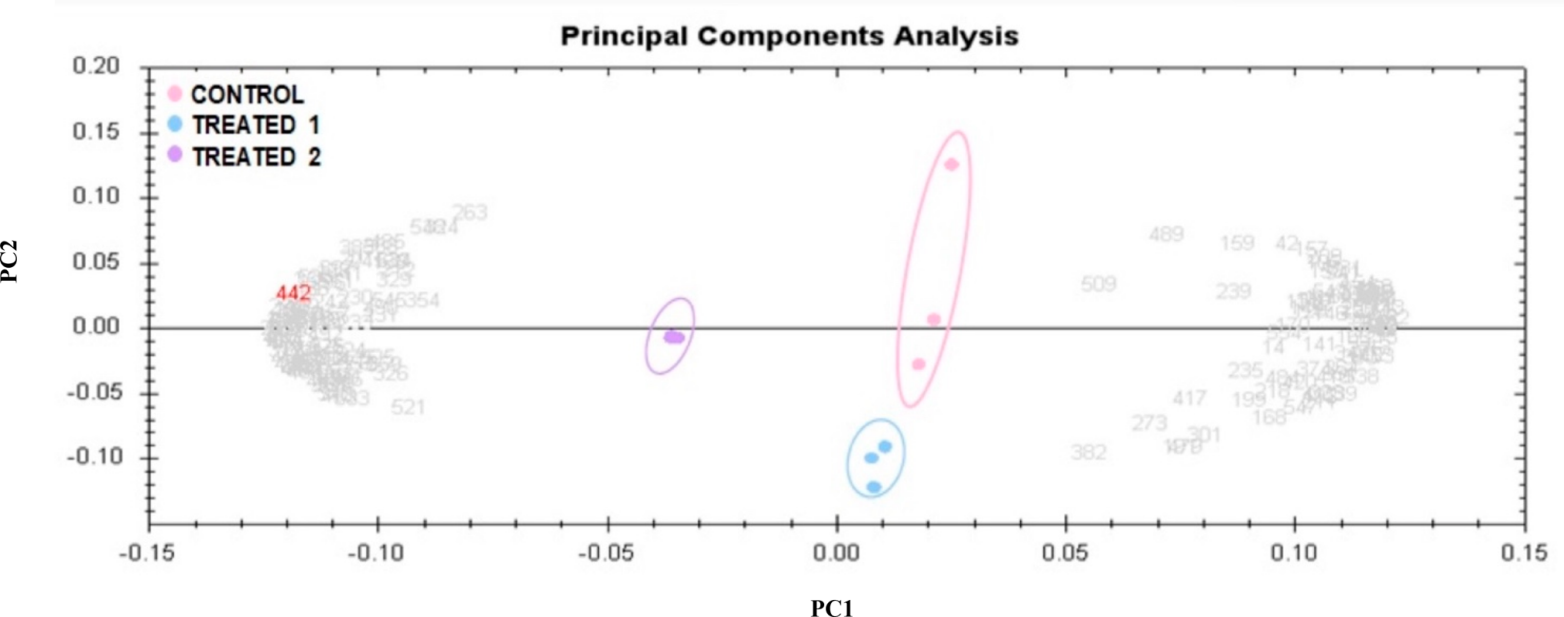
Principal component analysis of the proteomic data set. Pink dots are samples from the control group, blue dots are the samples from the JR-treated group (treatment 1), and purple dots are the samples from the MA-treated group (treatment 2). Together these explained 79.84% of the variability of selected spots. Colored dots and numbers are the representation of all spots in the gels.

**Figure 5:**
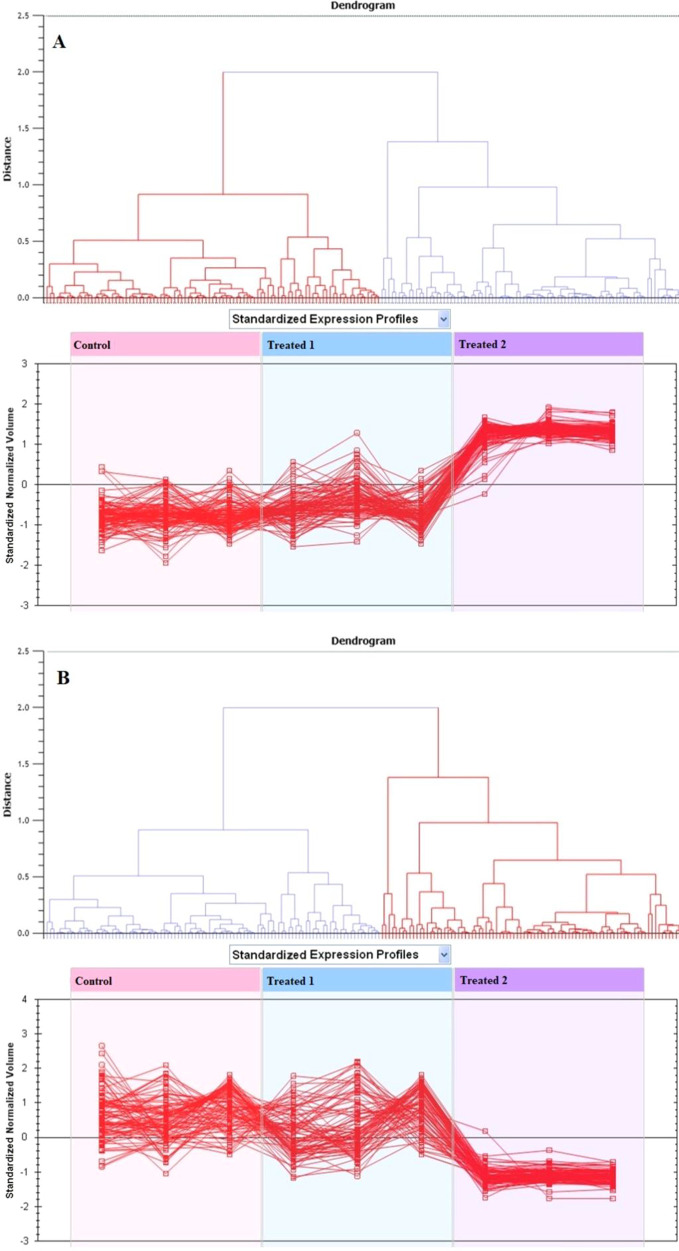
Expression profiles are divided into clusters of expression forms, showing the number of spots in each cluster. Each line displays the standardized abundance of a spot across all gels and belongs to one of the clusters generated by hierarchical cluster assessment. The spots with higher abundance were the 48 upregulated proteins in MA-treated (treated 2) (A). The spots with decreased abundance were the 11 upregulated proteins in JR-treated (treated 1) (B) (Progenesis Same Spots).

**Figure 6:**
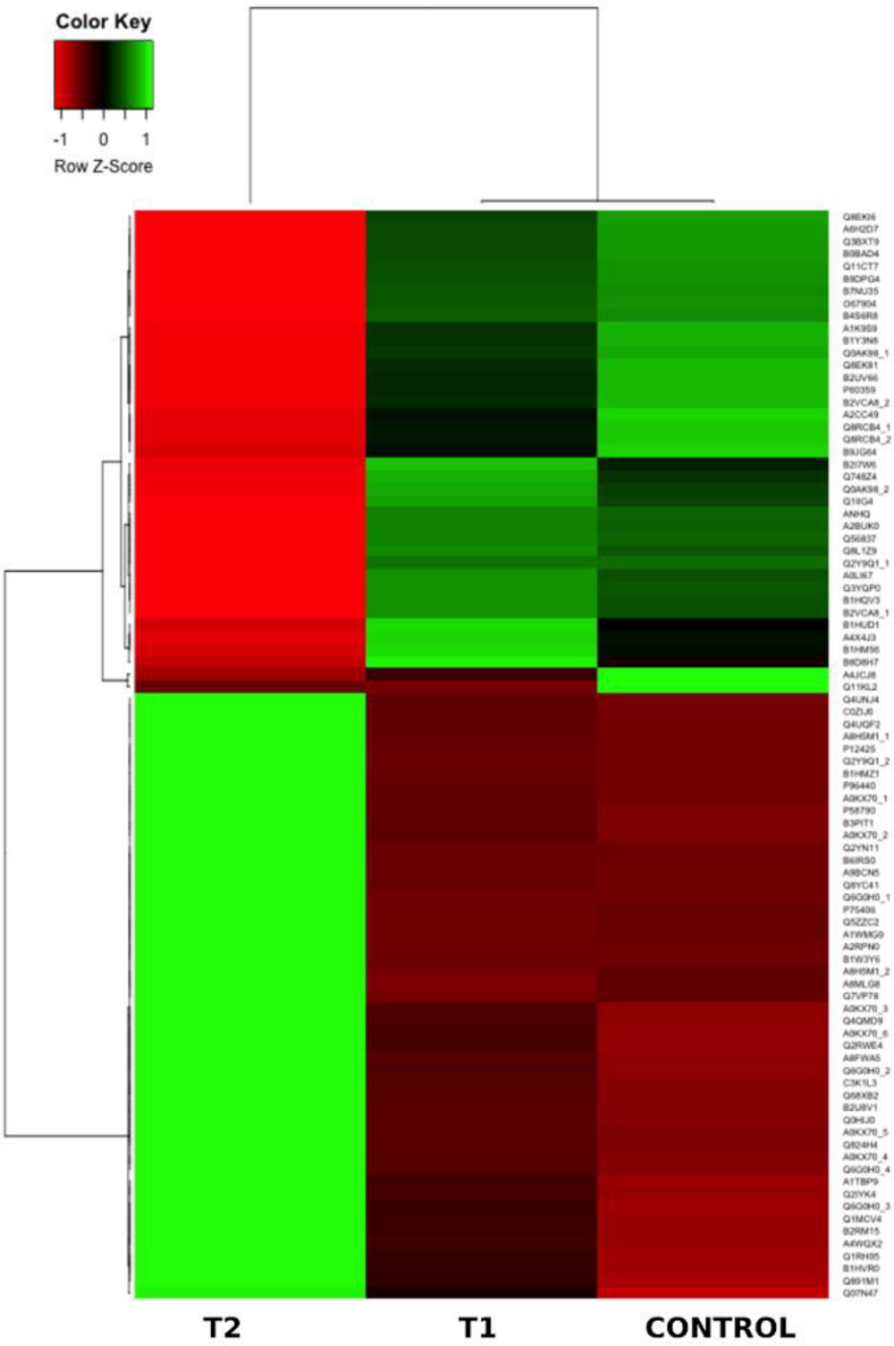
Heatmap representation of the differentially expressed protein spots from the control, JR-treated (T1), and MA-treated (T2) samples.

Principal component analysis (PCA) was performed to efficiently represent and correlate variables related to the features of the 190 differentially expressed proteins from the preparative gel shown in Figure 3. PCA confirmed the significant changes in abundance (*p* < 0.05 by ANOVA), as noted by MS, and the three groups were markedly clustered from one another based on the abundance of proteins, with 79.84% as the cutoff score (Figure 4). Based on hierarchical clustering analysis, differentially abundant spots revealed clusters of expression patterns, as shown in Figure 5. The clustering pattern showed that the variation in protein abundance for selected spots between control, JR-treated, and MA-treated samples (Figure 5A,B) differed significantly. Moreover, all 88 proteins detected by MS were used to create a heatmap, with shades of red demonstrating high expression levels and green indicating low expression levels. The heatmap (Figure 6) revealed that the most recognized proteins had upregulated expression patterns when comparing the control samples to the JR-treated and MA-treated samples. In addition, a more remarkable proteomic profile change was apparent in the MA-treated samples than in the JA-treated samples.

### 3.4 STRING Analysis

**Figure 7:**
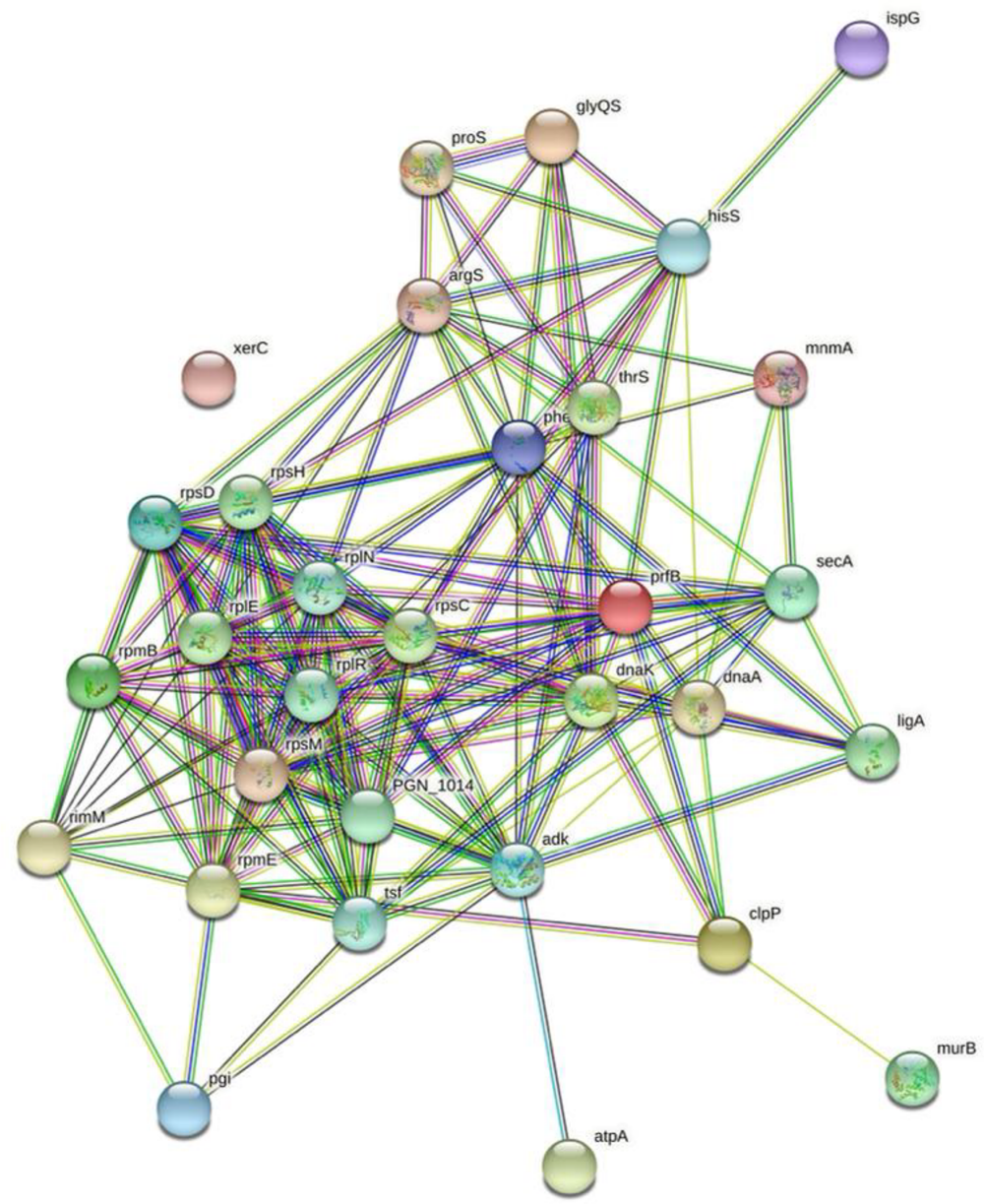
Protein–protein interaction network of the differentially expressed proteins between the control, JR-treated, and MA-treated samples using STRING v11.0 (https://string-db.org/). Nodes and edges are displayed, and an increasing number of edges indicate greater interactions.

The interaction of differently expressed protein networks was evaluated using bioinformatic assessment by STRING v11.0 (Figures 7 and S1).

## 4 Discussion

### 4.1 Response of *P. gingivalis* upon Treatment with *J. regia* (JR) and *M. alternifolia* (MA)

The adaptation mechanism of bacteria to overcome and survive antimicrobial treatment poses a critical problem for the pharmaceutical industry in providing clinically efficacious antibacterial drugs. To facilitate the drug-discovery process, proteomics can be applied to understanding the responsive mechanisms of bacteria used to evade antimicrobial cytotoxicity and sheds light on the mechanisms of action of many new antibacterial drug candidates. Furthermore, drugs of plant origin may be a more efficacious and safer alternative to the known chemical agents for developing new antibiotics.

Earlier studies have identified metabolites from JR and MA. Naphthoquinones are the major phenolic group in *Juglans regia*, and juglone is the characteristic compound.^23^ In addition, terpinen-4-ol and α-terpineol are key antimicrobial metabolites in tea tree oil.^14^ Various studies addressed the possible mechanism of such compounds as antimicrobial agents where juglone showed the ability to suppress the biofilm formation, and molecular docking analysis indicated its ability to bind in the active site of Sortase A and therefore predicted it as a strong enzyme inhibitor.^24^ Furthermore, a stable interface was noted when terpinen-4-ol was docked to the autolysin receptor as a microbial target.^25^

Identifying the proteomic profile of microbes treated with natural agents compared to the untreated control helps to identify their mode of action, regulated proteins, active genes, and transcriptional regulatory mechanisms during treatment, which may facilitate the design of new drugs to preclude antimicrobial resistance. Furthermore, *P. gingivalis* initiates the production of several virulence factors, such as proteases (gingipains), assisting in breaking down of the host immune proteins such as IL-1 CD 14; lipopolysaccharides, cell surface lipopolysaccharides which help to resist the host complement system; and short-chain fatty acids which promote and induce host cells to undergo apoptosis.^26^ As such, antibacterial and cytotoxic mechanisms affecting multiple metabolic pathways and proteins would lead to abated pathogenesis and virulence factors, whereas adaptive and resistive responses would lead to augmented pathogenesis and virulence factors.

The JR and MA plant extracts were assessed and identified as active agents against the periodontal pathogen *P. gingivalis*. The effect of plant molecules and their inhibitory effect on the growth of oral pathogenic bacteria and dental plaque formation have been well-reviewed.^27^ Furthermore, various studies have validated the effect of JR and MA on improving dental health and oral hygiene by suppressing the growth of *P. gingivalis*.^16,28^ Further, the proteomic study was done in a trial to detect any differences in *P. gingivalis* metabolism when treated with JR or MA extracts which might support the use of herbal extracts as safe antibiotics agents where the development of resistance genes could be rare.^29^ One postulated mechanism by which herbal-induced resistance could be rare is their ability to effectively modulate host processes by interacting, binding, and modifying proteins, preventing and interfering with the host–pathogen protein–protein interactions and hence dismantling the communication system essential for effective pathogenicity. Eighty-eight proteins were detected, from which 11 proteins were upregulated in JR-treated bacteria and 48 were upregulated in MA-treated bacteria, of which both treatments commonly upregulated 10 proteins. Such proteins were four 50S ribosomal proteins, cell division topological specificity factor, UPF0225 protein Shewana3_2159, Arginine-tRNA ligase, ATP-dependent Clp protease proteolytic subunit, 4-hydroxy-3-methylbut-2-en-1-yl diphosphate synthase (flavodoxin), and Ribose-5-phosphate isomerase A. Furthermore, seven proteins were commonly downregulated in both treatments: protein translocase subunit SecA, 30S ribosomal protein S13, ATP synthase subunit alpha, NAD(P)H dehydrogenase (quinone), phosphoribosyl-ATP pyrophosphatase, ribosome maturation factor RimM, and Acetyl-coenzyme A carboxylase carboxyl transferase subunit beta. Among the identified proteins, some subunits were found in more than one spot on the preparative gel, signifying slight variations in protein sequence and structure, including post-translational modifications, alternative splice transcripts, and alternative enzyme cleavage products.

Our results show that the *in vitro* antibacterial effect of the MA extract on *P. gingivalis* was not significantly different from that of the JR extract. In addition, both treatments impacted the bacterial proteome, with several common differentially expressed proteins (10 upregulated and 7 downregulated), most likely contributing to cytotoxicity via similar mechanisms. However, a more significant fold change in all the common differentially expressed proteins was observed as a consequence of MA treatment than JR treatment. Furthermore, similarities in regulating some of the proteins suggest some overlap in the mode of action for JR and MA extracts.

### 4.2 Ribosomal Proteins

50S ribosomal protein L14, 50S ribosomal protein L5, 50S ribosomal protein L28, and 50S ribosomal protein L31 were upregulated in *P. gingivalis* as a result of JR and MA treatments. Ribosomal proteins are a structural constituent of the ribosome, facilitate the binding of rRNA, and are essential for the translational process. Antibacterial agents are known to promote the formation of ROS in bacteria^30^ to mediate cytotoxic effects; increased expression of the ribosomal proteins allows augmented translational responses, possibly producing proteins that mitigate the treatment-induced ROS and allow adaptation to the stressful environment. Reference (19) noted the upregulation of five ribosomal proteins from *E. coli* treated with plant extracts. Reference (31) reported that changes in proteins in response to harsh conditions might occur in a microbial cell for cell growth and development adjustment. One study identified increased transcriptional activity of a gene responsible for a ribosomal protein in *P. gingivalis* exposed to polyphosphate.^32^ Upregulation of gene transcription of various ribosomal proteins was noted when *Clostridium difficile* was treated with clindamycin and amoxicillin and when *Streptococcus pneumoniae* R6 was treated with erythromycin, chloramphenicol, tetracycline, and puromycin.^33,34^ On the other hand, 30S ribosomal protein S13, responsible for RNA/tRNA binding, was downregulated as an effect of both JR and MA extracts. The downregulation of this protein was associated with an antimicrobial effect of placental extracellular vesicles on group B *Streptococcus*.^35^

### 4.3 Arginine-tRNA Ligase

Transfer RNAs are also important targets for synthetic and natural antibiotics.^36^ Aminoacyl-tRNA synthetases are a group of 20 amino acid enzymes that, during the translational process, link tRNAs to their corresponding amino acids for protein buildup.^37^ Arginine-tRNA ligase protein is an aminoacyl tRNA synthetase essential for protein synthesis that plays a vital role in cell viability and growth.^38^ Since the Arginine-tRNA ligase is a key target for antimicrobial agents,^36^ the observed increase in Arginine-tRNA ligase protein expression in response to drug treatment may indicate an adaptational process to maintain basal enzyme levels allowing normal growth processes to be unaffected.^36^

### 4.4 ATP-Dependent Clp Protease Proteolytic Subunit

ATP-dependent proteolytic Clp enzymes are essential in maintaining normal microbial growth and ensuring healthy cellular functions by degrading misfolded proteins and removing dysfunctional proteins, thus reducing the level of cellular stress^39^ akin to cellular autophagy in eukaryotic cells. In addition, in some bacteria, such as *Salmonella typhimurium* and *Listeria monocytogenes*, the ClpP has been linked with the expression of virulence genes.^40^ In periodontal disease, *P. gingivalis* is a frequently encountered pathogen that utilizes Clp proteases in plaque biofilm formation for increased pathogenicity and virulence.^41^ The ATP-dependent Clp protease proteolytic subunit is an important biofilm regulator.^42^ As such, an increase in expression of this protein on treatment with JR and MA may be an adaptive mechanism to evade intracellular damage caused by the antimicrobial agents and to promote a virulence-driving extracellular microenvironment.

### 4.5 4-Hydroxy-3-methylbut-2-en-1-yl Diphosphate Synthase (Flavodoxin)

Flavodoxins are redox-active proteins responsible for electron transfer in a bacterial cell. In addition, flavodoxin is essential in the non-mevalonate isoprenoid/terpenoid synthesis pathway. Isoprenoids are essential for regulating normal cellular function and survival. They are important in protein prenylation and function; as such, they can regulate gene expression and make up active metabolites required within the cell.^43^ Therefore, they play an important role in many metabolic pathways and are an antimicrobial target in some bacteria.^44^*H. pylori* treatment with metronidazole caused a decrease in Flavodoxin expression, thus suggesting that flavodoxin could be a potential antibacterial target for this bacterium.^45^ In the current study, we found Flavodoxins were overexpressed on *P. gingivalis* treatment with JR and MA extracts, which could be another mechanism for virulence and adaptation.

### 4.6 Protein Translocase Subunit SecA

SecA plays an essential role in the protein translocation process and acts as an ATPase providing energy for Sec-dependent protein translocation in bacterial cells. The inhibition of SecA leads to antibacterial effects, and thus, it has been suggested as a potential antibacterial drug target.^46^ Furthermore, SecA is vital for bacterial pathogenesis since it releases virulence factors, toxins, and other essential proteins, hence playing a role in survival.^47^ Accordingly, treatment with JR and MA extracts led to decreased protein translocase subunit SecA expression, identifying one mode of action by which these herbal agents might elicit their antibacterial effects.

### 4.7 ATP Synthase Subunit Alpha

ATP synthase produces ATP from ADP, and subunit alpha is the regulatory unit. The downregulation of ATP synthase subunits would suppress the normal energy-dependent metabolic processes and growth of *P. gingivalis*. In addition, ATP synthase subunits have been reported as an antibacterial target in *Pseudomonas aeruginosa* and others.^48,49^ In addition, a severe reduction in ATP synthesis in *Enterococcus faecalis* and *E. faecium* was observed when treated with terpenoids from *Salvia tingitana*.^50^ Our findings suggest that JR or MA contain phyto-molecules that could target the production of ATP and thus impart effects on energy-dependent metabolic pathways in *P. gingivalis*.

### 4.8 NAD(P)H Dehydrogenase (Quinone)

In most organisms, reduced NADH and quinones are vital in the bacterial respiratory system as electron and proton carriers.^51^ NAD(P)H usually is present in the inner cytoplasmic membrane and is a reducing agent which drives anabolic reactions, such as fatty acid synthesis and DNA. As such, it is essential for synthesizing cellular components, a prerequisite for bacterial growth and replication.^52^ In addition, NADPH helps in maintaining a redox balance within the cell and thus may protect the cell against ROS-induced toxicity.^53^ Reduced protein levels in JR and MA-treated *P. gingivalis* suggest the possibility of such herbal agents as inhibitors of respiratory enzymes as a mode of their antibacterial action. An approved NADPH dehydrogenase inhibitor, Polymyxins, is currently used as an antimicrobial for *E. coli* infections.^54^

### 4.9 Ribosome Maturation Factor RimM

RimM is known to be involved in the maturation of the 30S ribosomal subunit.^55^ Inhibiting RimM or reducing its expression would lead to immature and dysfunctional ribosomal protein, consequently leading to a translation defect.^56^*P. gingivalis* treatment with JR or MA extracts led to a decreased expression of RimM, which would inevitably prevent the translation of essential proteins required for survival, growth, and pathogenesis.

### 4.10 Acetyl-coenzyme A Carboxylase Carboxyl Transferase Subunit Beta

Acetyl coenzyme A (acetyl-CoA) carboxylase in bacteria is a multisubunit heterohexamer enzyme essential for bacterial growth and development. It catalyzes fatty acid biosynthesis by an irreversible reaction forming malonyl-CoA by carboxylation of acetyl-CoA,^57^ and lipid biosynthesis is important for the pathogenesis of *P. gingivalis* and virulency.^26^ Furthermore, the primary mode of action of moiramide B antibiotics is targeting acetyl coenzyme A carboxylases. Therefore, the downregulation of acetyl coenzyme A on treatment with JR and MA suggests that these extracts can inhibit the first essential step in lipid biosynthesis, affecting cellular activity and growth by modulating the protein expression of this essential enzyme.

### 4.11 Bioinformatics Analysis

The interaction network of the differentially expressed protein from *P. gingivalis* identified 31 of the 32 proteins as having common pathways and functional networks, indicating a tightly regulated network of essential proteins through which the herbal extracts elicit their cytotoxic effects and by which *P. gingivalis* actively resists cytotoxic effects. In terms of drug targets, the strong interactions indicated by the significant number of edges to most nodes identify that targeting only one protein from this network might not give the desired antibacterial efficacious effect. Instead, a better approach would be to use multiple targets. Hence, herbal extracts may be the way forward due to the array of phytochemicals present in the extract having their unique targets involving various interconnected processes required for cell growth differentiation, repair, and pathogenicity. Indeed, disrupting interactions within metabolic pathways is a strategy for efficacy in antibiotics.^58^ Metabolic instability appears to be the mode of action of JR and MA extract as antimicrobial agents against *P. gingivalis*. An earlier study on *P. gingivalis* treated with nicotine and cotinine found proteins related to metabolism and protein biosynthesis.^59^

### 4.12 Conclusion

Our work discovers overexpressed proteins fundamental in the translational process, critical proteins involved in energy production and biochemical pathways and central in protein regulation, function, repair, and removal. Consequently, we reveal how *P. gingivalis* organizes its proteome to adapt to the impact of environmental stresses induced by antibacterial herbal extracts JR and MA, allowing it to resist, heal, grow, and replicate and promoting its pathogenesis. Thus, the overexpressed proteins may be essential in mechanisms promoting antimicrobial resistance. As such, these proteins and their related pathways may be critical targets for future efficacious antibacterial drugs combating resistive mechanisms in *P. gingivalis*. Additionally, we identified downregulated proteins: ATP synthase subunit alpha, vital for energy formation; NADPH dehydrogenase, an N terminal reducing agent essential in many metabolic pathways; ribosome maturation factors, resulting in immature ribosomes; and those involved in fatty acid biosynthesis. The down-regulated proteins distinguish the mode of antibacterial action of the herbal extracts, identify precise antimicrobial targets, and can be used as a measure of efficacy. We suggest further experiments comparing the proteomic profiles generated here with expression profiles from current antibiotics used against *P. gingivalis*. In addition, variations in expression patterns may be expected related to the dose or concentration and length of treatment applied. In addition, it is important to consider the efficacy of prospective antibacterial agents since using mild agents may give the bacteria the needed time and opportunity to switch on their adaptive mechanisms and completely offset the intended antibacterial effects before any significant harm beyond unrepairable damage is made. This introduces another level of complexity when testing antibacterial agents. Furthermore, applying plant extracts as antimicrobial agents is a good alternative to the known chemical agents, and it may also help prevent *P. gingivalis*-associated chronic systemic diseases and periodontitis.

## Supporting Information Available

Supplementary data, raw data for reference gel, and string raw data (PDF)
