# Supplementary material for: Proteomic Profiling Reveals Cytotoxic Mechanisms of Action and Adaptive Mechanisms of Resistance in Porphyromonas gingivalis: Treatment with Juglans regia and Melaleuca alternifolia
Source: ACS Omega. 2023 Mar 28;8(14):12980–91. doi: 10.1021/acsomega.3c00168 (PMC10099446; doi:10.1021/acsomega.3c00168)
Supplement: Supplementary file 1 — ao3c00168_si_001.pdf [file ao3c00168_si_001.pdf]

Supporting Information

Proteomic Profiling Reveals Cytotoxic Mechanisms of Action and Adaptive Mechanisms of Resistance in *Porphyromonas Gingivalis*: Treatment with *Juglans Regia* and *Melaleuca Alternifolia*

Afrah E. Mohammed[1#], Kawther Aabed[1#], Hicham Benabdelkamel[2], Ashwag Shami[1], Modhi Alotaibi[1], Mona Al-enazi[1], Assim A. Alfadda[2], Ishrat Rahman[3*]

[1] Department of Biology, College of Science, Princess Nourah bint Abdulrahman University, P.O. Box 84428, Riyadh 11671, Saudi Arabia

[2]Proteomics Unit, Obesity Research Center, Department of Medicine, College of Medicine, King Saud University, Riyadh 11461, Saudi Arabia

[3]Department of Basic Dental Sciences, College of Dentistry, Princess Nourah bint Abdulrahman University, P.O. Box 84428, Riyadh 11671, Saudi Arabia

# Authors contributed equally

* Correspondence should be addressed IMRahman@pnu.edu.sa

Figure S1. The figure shows the different gene count obtained from STRING functional analysis
Table S1. Mass spectrometry list of significant differentially abundant proteins between control, JR-treated and MA -treated samples identified in 10 samples, using 2D-DIGE. Protein name, accession number, Mascot score, MS % coverage, protein MW and pI values are listed according to Uniprot database

Supplementary data:

Figure S1: The figure shows the different gene count obtained from STRING functional analysis

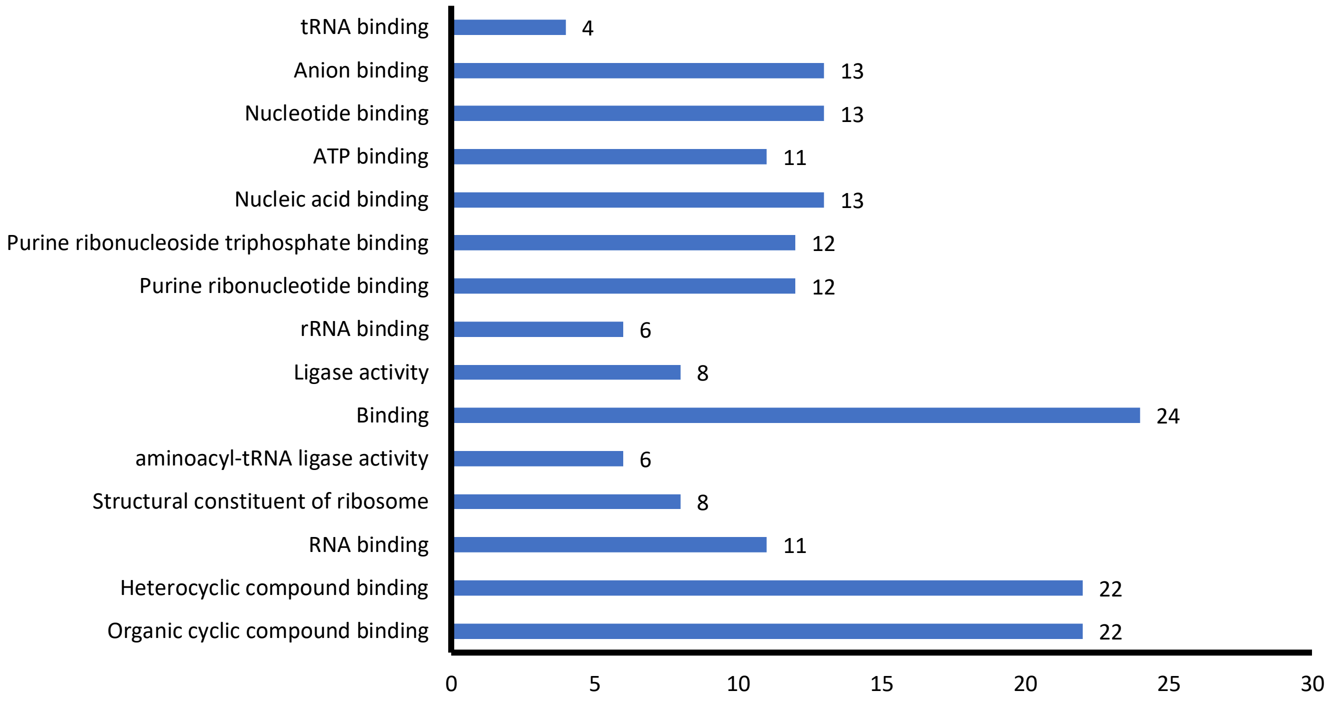

Table S1: Mass spectrometry list of significant differentially abundant proteins between control, JR-treated and MA -treated samples identified in 10 samples, using 2D-DIGE. Protein name, accession number, Mascot score, MS % coverage, protein MW and pI values are listed according to Uniprot database

SI No.	Spot No[a]	Accession Nob	Protein Name	MASCOT ID	Pi[c]	MW[d]	Cov%	Score
1	442	P75406	Uncharacterized protein MPN_375	Y375_MYCPN	9.95	15193	29	59
2	274	P12425	Glutamine synthetase	GLNA_BACSU	5.05	50531	22	74
3	261	C3K1L3	Histidine-tRNA ligase	SYH_PSEFS	5.61	48081	19	77
4	257	B2U8V1	Peptide chain release factor 2	RF2_RALPJ	4.94	41370.	16	62
5	187	A0KX70	UPF0225 protein Shewana3_2159	Y2159_SHESA	7.59	19115	35	64
6	248	Q4UQF2	ATP synthase subunit alpha	ATPA_XANC8	5.32	55404	19	66
7	373	A0KX70	UPF0225 protein Shewana3_2159	Y2159_SHESA	7.59	19115	35	70
8	202	Q3YQP0	Threonine--tRNA ligase	SYT_EHRCJ	6.89	73843	18	63
9	538	B4S6R8	Transcriptional regulator MraZ	MRAZ_PROA2	8.73	17890	55	66
10	162	Q0AK98	UPF0102 protein Mmar10_3014	Y3014_MARMM	11.65	14627	37	59
11	413	C0ZIJ0	50S ribosomal protein L14	RL14_BREBN	9.94	13147	59	69
12	355	B3PIT1	ATP synthase subunit b	ATPF_CELJU	5.97	17199	39	74
13	339	B2VCA8	ATP synthase subunit b	ATPF_ERWT9	5.40	17306	56	83
14	536	Q6G0H0	ATP synthase subunit b 2	ATPF2_BARQU	5.17	18567	53	102
15	166	B6IRS0	30S ribosomal protein S8	RS8_RHOCS	10.06	14774	49	66
16	475	Q5ZZC2	UPF0178 protein lpg0089	Y089_LEGPH	9.59	16537	40	67
17	299	A0KX70	UPF0225 protein Shewana3_2159	Y2159_SHESA	7.59	19115	48	76
18	161	Q8RCB4	Protein translocase subunit SecA	SECA_THETN	6.05	103248	14	63
19	102	Q56837	2-hydroxypropyl-CoM lyase	XECA_XANP2	5.59	41835	30	59
20	390	A0AJK7	Aspartyl/glutamyl-tRNA(Asn/Gln) amidotransferase subunit B	GATB_LISW6	5.26	53296	25	60
21	436	Q68XB2	Ribosomal large subunit pseudouridine synthase C	RLUC_RICTY	9.54	34697	23	69
22	260	A8FWA5	Cell division topological specificity factor	MINE_SHESH	5.63	10016	45	69
23	171	Q6G0H0	ATP synthase subunit b 2	ATPF2_BARQU	5.17	18567	30	66
24	225	B2VCA8	ATP synthase subunit b	ATPF_ERWT9	5.40	17306	43	82
25	440	P58790	1-(5-phosphoribosyl)-5-[(5-phosphoribosylamino)methylideneamino] imidazole-4-carboxaide isomerase	HIS4_AGRT5	4.68	26125	29	71
26	211	Q748Z4	30S ribosomal protein S3	RS3_GEOSL	9.97	23939	38	79
27	473	A9BCN5	50S ribosomal protein L5	RL5_PROM4	10.24	19992	37	67

28	96	Q8EKI6	Sulfur carrier protein FdhD	FDHD_SHEON	6.60	30838	14	58
29	347	A0LI67	DNA ligase	DNLJ_SYNFM	6.12	74411	12	75
30	459	A0KX70	UPF0225 protein Shewana3_2159	Y2159_SHESA	7.59	19115	35	61
31	245	Q6G0H0	ATP synthase subunit b 2	ATPF2_BARQU	5.17	18567	37	74
32	183	A2CC49	30S ribosomal protein S13	RS13_PROM3	11.33	13702	40	64
33	177	A8H5M1	tRNA-specific 2-thiouridylase MnmA	MNMA_SHEPA	4.95	41646	24	61
34	375	Q07N47	Serine--tRNA ligase	SYS_RHOP5	5.54	52688	26	59
35	397	A8H5M1	tRNA-specific 2-thiouridylase MnmA	MNMA_SHEPA	4.95	41646	24	60
36	242	A1TBP9	Glycine--tRNA ligase	SYG_MYCVP	5.51	53360	14	58
37	385	A8MLG8	30S ribosomal protein S4 A	RS4A_ALKOO	9.53	23540	28	59
38	178	A0KX70	UPF0225 protein Shewana3_2159	Y2159_SHESA	7.59	19115	35	63
39	510	Q8RCB4	Protein translocase subunit SecA	SECA_THETN	6.05	103248	25	82
40	499	Q2IYK4	UDP-N-acetylenolpyruvoylglucosamine reductase	MURB_RHOP2	6.24	32697	26	65
41	384	B2RM15	50S ribosomal protein L28	RL28_PORG3	10.06	9118	53	62
42	264	Q8L1Z9	Glucose-6-phosphate isomerase	G6PI_BARHE	6.84	62734	23	61
43	199	B1HUD1	Chaperone protein DnaK	DNAK_LYSSC	4.63	65320	28	66
44	378	A0KX70	UPF0225 protein Shewana3_2159	Y2159_SHESA	7.59	19115	35	69
45	412	A2BUK0	Probable aspartoacylase	ASPA_PROM5	8.57	34492	21	70
46	344	Q1RH05	50S ribosomal protein L31	RL31_RICBR	9.34	8896	56	61
47	410	Q2Y9Q1	Cytochrome c biogenesis ATP-binding export protein CcmA	CCMA_NITMU	6.06	22320	32	65
48	561	Q2Y9Q1	Cytochrome c biogenesis ATP-binding export protein CcmA	RS8_LYSSC	9.61	14858	65	98
49	320	Q4UNJ4	Probable tRNA-dihydrouridine synthase	DUS_RICFE	6.97	35556	26	64
50	441	Q8YC41	Putative binding protein BMEII0691	Y3691_BRUME	6.55	69344	20	64
51	259	B1Y3N8	Heat-inducible transcription repressor HrcA	HRCA_LEPCP	5.57	37130	10	63
52	349	Q3BXT9	ATP-dependent protease ATPase subunit HslU	HSLU_XANC5	5.36	50719	18	65
53	201	A1WMG9	Phenylalanine--tRNA ligase alpha subunit	SYFA_VEREI	6.22	38955	33	67
54	280	Q1MCV4	Urease accessory protein UreD	URED_RHIL3	9.47	29300	24	60
55	101	B7NU35	tRNA/tmRNA (uracil-C(5))-methyltransferase	TRMA_ECO7I	6.08	42231	19	58
56	374	Q0AK98	UPF0102 protein Mmar10_3014	Y3014_MARMM	11.65	14627	50	65

57	346	P60359	UPF0297 protein SA1445	Y1445_STAAN	4.75	10297	48	61
58	136	A6H2D7	ATP synthase subunit alpha	ATPA_FLAPJ	5.36	56663	28	80
59	444	Q6G0H0	ATP synthase subunit b 2	ATPF2_BARQU	5.17	18567	37	62
60	83	A1K9S9	NAD(P)H dehydrogenase (quinone)	WRBA_AZOSB	6.59	21071	37	72
61	167	B1HMZ1	Elongation factor G	EFG_LYSSC	5.00	76556	25	100
62	547	Q11CT7	Protease HtpX homolog	HTPX_MESSB	7.98	33770	23	60
63	448	Q824H4	Arginine--tRNA ligase	SYR_CHLCV	5.79	63869	15	60
64	331	B9JG64	Phosphoribosyl-ATP pyrophosphatase	HIS2_AGRRK	5.17	11351	57	70
65	241	P96440	Exopolysaccharide II synthesis transcriptional activator ExpG	EXPG_RHIME	6.44	21828	30	63
66	239	A4JCJ8	Ribosome maturation factor RimM	RIMM_BURVG	5.30	24191	22	65
67	417	B8D8H7	Chromosomal replication initiator protein DnaA	DNAA_BUCA5	9.55	53240	16	60
68	170	B2I7W6	Proline--tRNA ligase	SYP_XYLF2	5.71	62810	15	67
69	401	Q0HIJ0	UPF0225 protein Shewmr4_2054	Y2054_SHESM	7.59	19118	35	68
70	498	B1HVR0	ATP-dependent Clp protease proteolytic subunit	CLPP_LYSSC	5.43	21671	47	79
71	386	Q7VP78	ATP-dependent Clp protease proteolytic subunit	CLPP_HAEDU	5.96	21716	40	68
72	362	Q891M1	Ribose import ATP-binding protein RbsA	RBSA_CLOTE	8.29	56749	15	60
73	484	B2UV66	50S ribosomal protein L18	RL18_HELPS	10.16	13443	39	58
74	188	Q4QMD9	Probable Fe(2+)-trafficking protein	FETP_HAEI8	6.11	10632	40	61
75	168	B1HQV3	GTP-sensing transcriptional pleiotropic repressor CodY	CODY_LYSSC	5.09	28929	24	68
76	301	B1HM56	ATP synthase subunit beta	ATPB_LYSSC	4.80	51243	30	85
77	235	A4X4J3	Elongation factor Ts	EFTS_SALTO	5.38	29776	23	69
78	389	Q8EK81	Elongation factor Tu 1	EFTU1_SHEON	5.13	43605	37	61
79	364	A4WQX2	Ribose-5-phosphate isomerase A	RPIA_RHOS5	4.86	27093	22	79
80	142	Q1IIG4	ATP synthase subunit b	ATPF_ACIBL	5.44	26005	35	71
81	534	Q2YN11	GTP pyrophosphokinase rsh	RSH_BRUA2	8.11	83975	21	71
82	548	A2RPN0	3-demethoxyubiquinol 3-hydroxylase	COQ7_HERSE	5.99	22962	37	59
83	509	Q11KL2	Acetyl-coenzyme A carboxylase carboxyl transferase subunit beta	ACCD_MESSB	6.06	33962	19	80
84	340	Q2RWE4	4-hydroxy-3-methylbut-2-en-1-yl diphosphate synthase (flavodoxin)	ISPG_RHORT	5.93	41216	19	60

85	553	B1W3Y6	Adenylate kinase	KAD_STRGG	4.88	23699	41	64
86	129	B9DPG4	Tyrosine recombinase XerC	XERC_STACT	9.37	34779	27	60
87	110	B0BAD4	Elongation factor Ts	EFTS_CHLTB	5.52	31005	24	63
88	105	O67904	Glutamyl-tRNA(Gln) amidotransferase subunit C	GATC_AQUAE	4.44	11208	31	59

[a] Spot numbers.

[b] Protein accession number for SWISSPROT Database.

[c] Theoretical isoelectric point.

[d] Theoretical relative mass.

[e] MASCOT score
